# Vitreoretinal lymphoma followed by systemic diffuse large B cell lymphoma

**DOI:** 10.1186/s12348-019-0177-8

**Published:** 2019-06-10

**Authors:** Kenneth C. Fan, Kimberly D. Tran, J. William Harbour, Sander A. Dubovy, Nimesh A. Patel, Thomas A. Albini

**Affiliations:** Department of Ophthalmology, Bascom Palmer Eye Institute, University of Miami Miller School of Medicine, 900 NW 17th St., Miami, FL 33136 USA

## Abstract

Vitreoretinal lymphoma as the presenting diagnosis in association with a systemic lymphoma without central nervous system involvement is exceedingly rare, and the classification of this condition is not well-established. Here, we describe a patient with intermittent blurry vision in the left eye for 2 years in the setting of a recent incidental diagnosis of diffuse large B cell lymphoma from an axillary lymph node biopsy. The diagnosis of panuveitis with an extensive exudative retinal detachment was made. The patient was treated with pars plana vitrectomy as well as systemic chemotherapy, intrathecal methotrexate, intravitreal methotrexate, and intravitreal rituximab with good post-operative outcomes.

## Introduction

Vitreoretinal lymphoma has classically been identified as a primary intraocular malignancy associated with primary central nervous system (CNS) lymphoma [1, 2]. When systemic lymphoma results in intraocular manifestations, the involvement is typically choroidal [1, 2]. However, recent reports of vitreoretinal lymphoma in the setting of systemic lymphoma have provided insight into the clinical presentation of non-primary vitreoretinal lymphoma (VRL) [3–6]. Rapid diagnosis, work-up, and treatment of secondary VRL (SVRL) are paramount as it can commonly masquerade as primary vitreoretinal lymphoma (PVRL) as well as retinitis, uveitis, and vasculitis [4, 6]. Here, we describe a case with a primary presentation of vitreous hemorrhage and underlying exudative retinal detachment secondary to an undiagnosed secondary vitreoretinal diffuse large B cell lymphoma (DLBCL) associated with a systemic DLBCL.

## Case report

A 54-year-old Korean male was referred to the uveitis service with the diagnosis of intermittent vitreous hemorrhage of unknown etiology in the left eye for 2 years. A week prior to presentation, the patient sustained trauma to his left arm resulting in a deep vein thrombosis. A thrombolectomy resulted in an incidental diagnosis of diffuse large B cell lymphoma, an activated B cell (ABC) subtype, from axillary lymph nodes. A review of systems revealed no history of severe infections, immunosuppression, nor intravenous drug use.

**Fig. 1:**
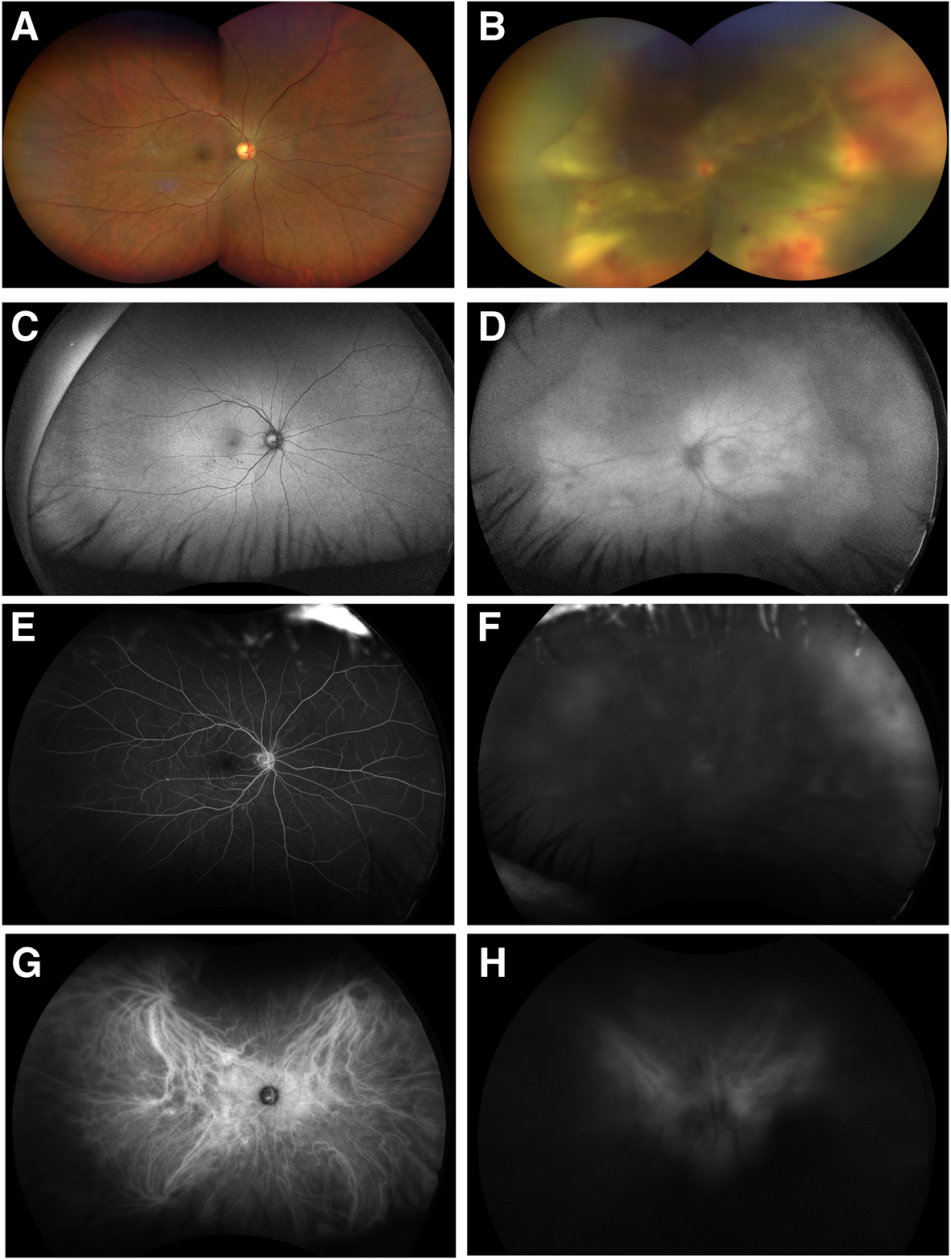
Fundus photographs, fundus autofluorescence, fluorescein angiography, and indocyanine green angiography of both eyes. Fundus photograph of the right eye was within normal limits, while the left eye demonstrated 4+ vitreous cell as well as dense vitreous haze with intraretinal submacular hemorrhage and exudative retinitis (**a**, **b**). Fundus autofluorescence demonstrated a normal right eye and hazy view with large patches of hyperautofluorescence of the left eye (**c**, **d**). Fluorescein angiography and indocyanine green of the right eye were normal, while the left eye demonstrated a hazy view with late peripheral hyperfluorescence/hypercyanescence (**e**–**h**)

**Fig. 2:**
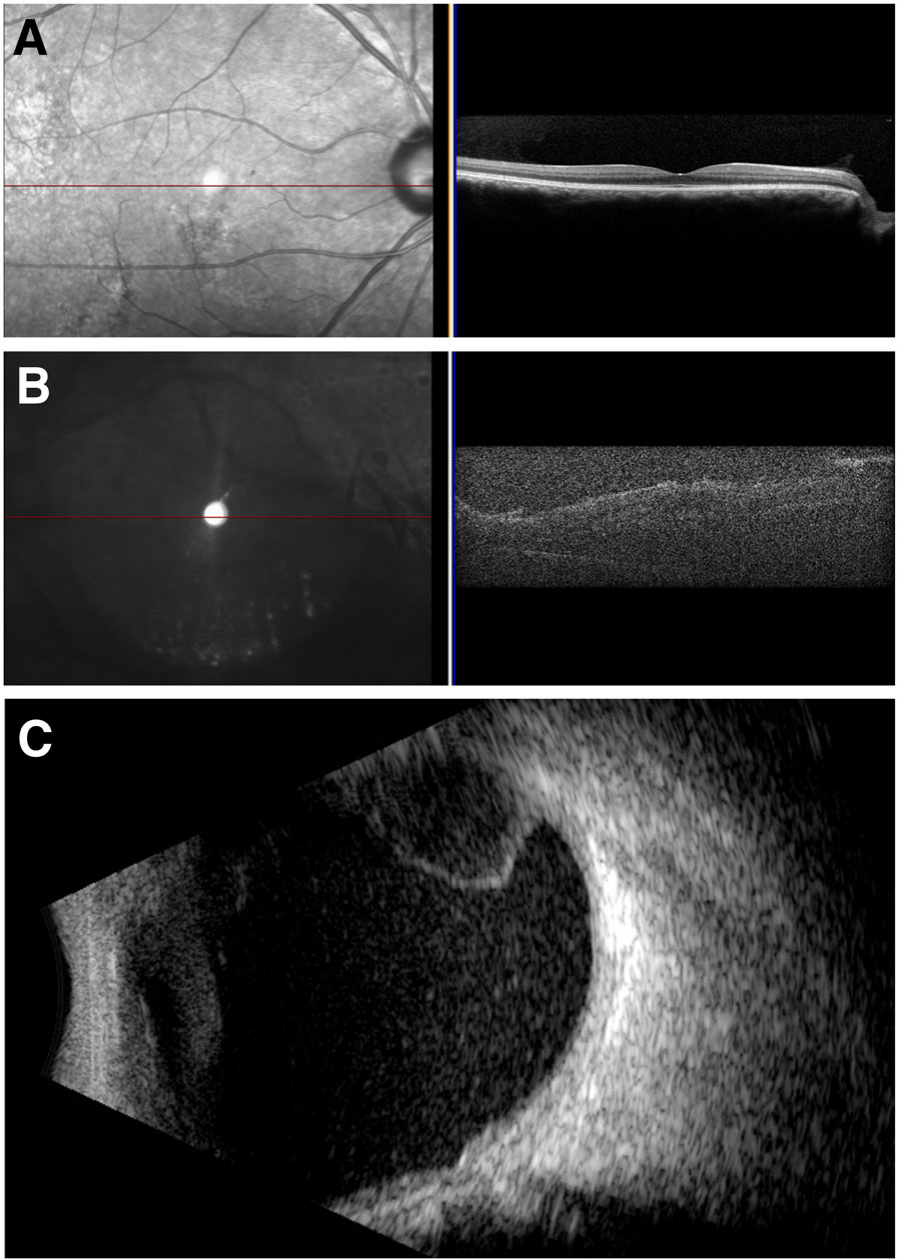
Optical coherence tomography and B-scan ultrasound of both eyes. Optical coherence tomography (OCT) of the right eye was normal (**a**). In the left eye, OCT demonstrated subretinal fluid and a limited quality scan due to vitreous haze (**b**). B-scan ultrasound noted a serous retinal detachment OS without any retinal breaks (**c**)

On presentation, the visual acuity was 20/20 in the right eye and 200E at 1 ft in the left eye. Examination of the left eye was notable for diffuse keratic precipitates and 1+ anterior chamber cell, as well as 4+ vitreous cell with intraretinal hemorrhage (Fig. 1a, b). Fundus autofluorescence, fluorescein angiography (FA), indocyanine green (ICG, Fig. 1e–h), and optical coherence tomography (OCT, Fig. 2a, b) of the left eye were limited due to the vitritis. B-scan ultrasound noted a serous retinal detachment without any retinal breaks (Fig. 2c). All imaging studies of the right eye were normal. Infectious and inflammatory work-up was negative, including normal CBC, Quantiferon, FTA-ABS, angiotensin-converting enzyme, and Toxoplasmosis IgG and IgM antibodies.

**Fig. 3:**
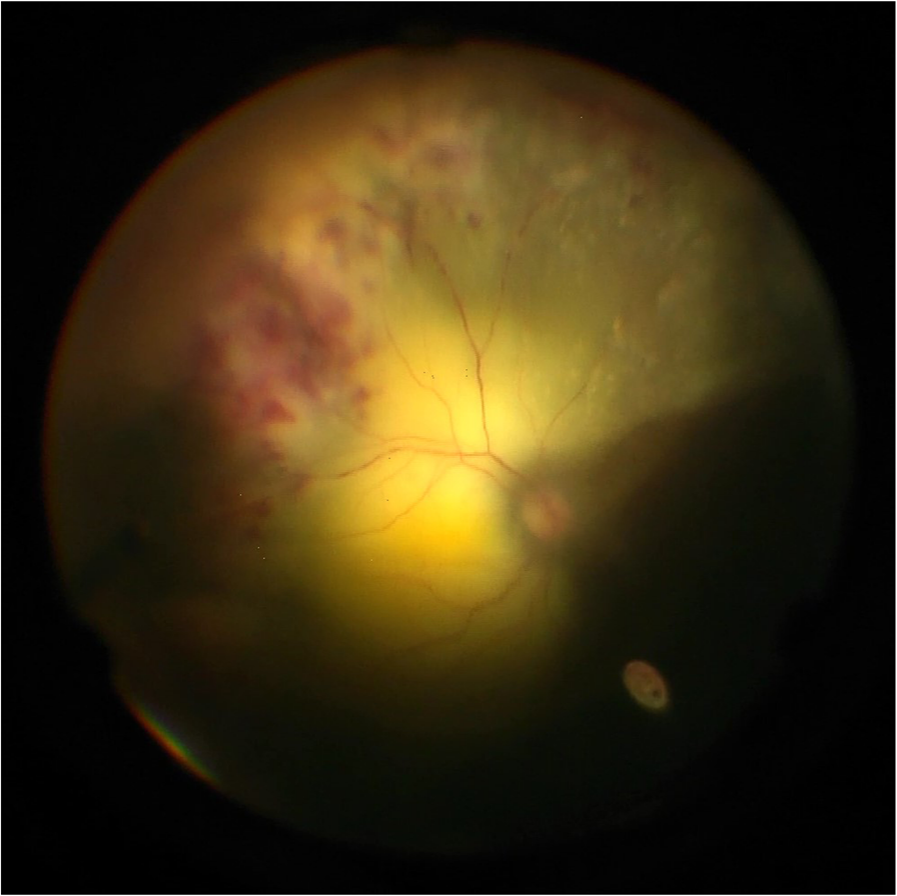
Intraoperative photo of diagnostic pars plana vitrectomy of the left eye. Intraoperatively, a large exudative retinal detachment with significant subretinal yellow-white deposits, sclerotic vessels, and intraretinal hemorrhages was noted. No retinal breaks were identified

A diagnostic and therapeutic pars plana vitrectomy was performed. An exudative retinal detachment with significant subretinal yellow-white deposits, sclerotic vessels, and intraretinal hemorrhages was noted (Fig. 3). No retinal breaks were identified, and no drainage of subretinal fluid was performed. Vitreous samples as well as the cassette were sent for microbiology, cytology, flow cytometry, pathology, and PCR.

**Fig. 4:**
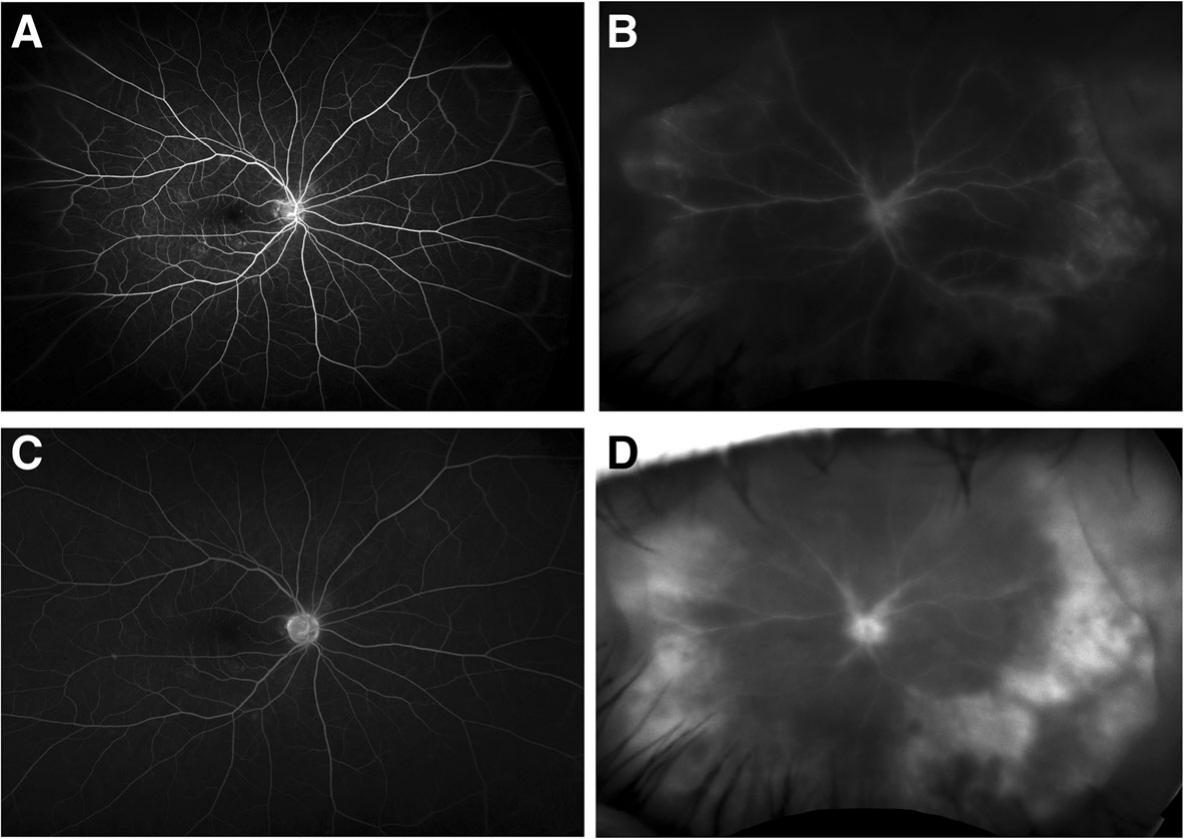
**a**–**d** Follow-up fluorescein angiography and autofluorescence of both eyes. Follow-up fluorescein angiography demonstrated a normal right eye and early perivascular and peripheral hyperfluorescence, with late diffuse hyperfluorescence and leakage in the left eye

**Fig. 5:**
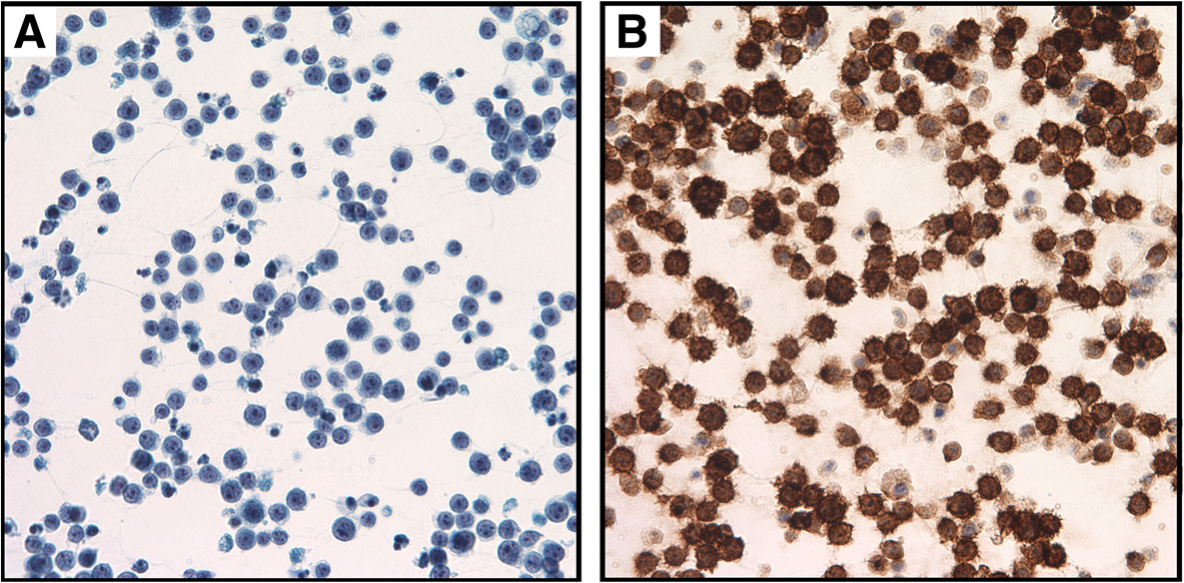
Pathology slides of vitreous biopsy taken from pars plana vitrectomy. Pathology specimens with Papanicolaou stain × 600 (**a**) and CD20 stain × 600 (**b**) demonstrating numerous atypical large CD20-positive lymphocytes with variably prominent nucleoli consistent with vitreoretinal large B cell lymphoma

Post-operatively, the patient was placed on topical steroids as well as oral valacyclovir. However, all vitreous microbiological studies were negative. Follow-up FA can be seen in Fig. 4. Pathology specimens demonstrated atypical CD20-positive lymphocytes consistent with large B cell lymphoma (Fig. 5). Histology and flow cytometry demonstrated a monoclonal B cell lymphoproliferative process, positive for CD19 and CD20 overexpression. Additional studies can be seen in Table 1 below. On further systemic work-up, orbital ultrasound, MRI of the head, orbit, face, and neck as well as whole body PET scan and cerebrospinal fluid analysis were normal.

**Table 1:** Left eye vitreous biopsy results

Flow cytometry	
Positive	B cell: CD19, CD20, lambda light chain restriction
Negative	CD5, CD10, CD23, CD34, CD38, CD45T cell: CD2, CD3, CD4, CD5, CD7, CD8, HLA-DR, G1
PCR genetic clonality test	
Positive	B cell monoclonality (IGH rearrangement)
Negative	T cell monoclonality

**Fig. 6:**
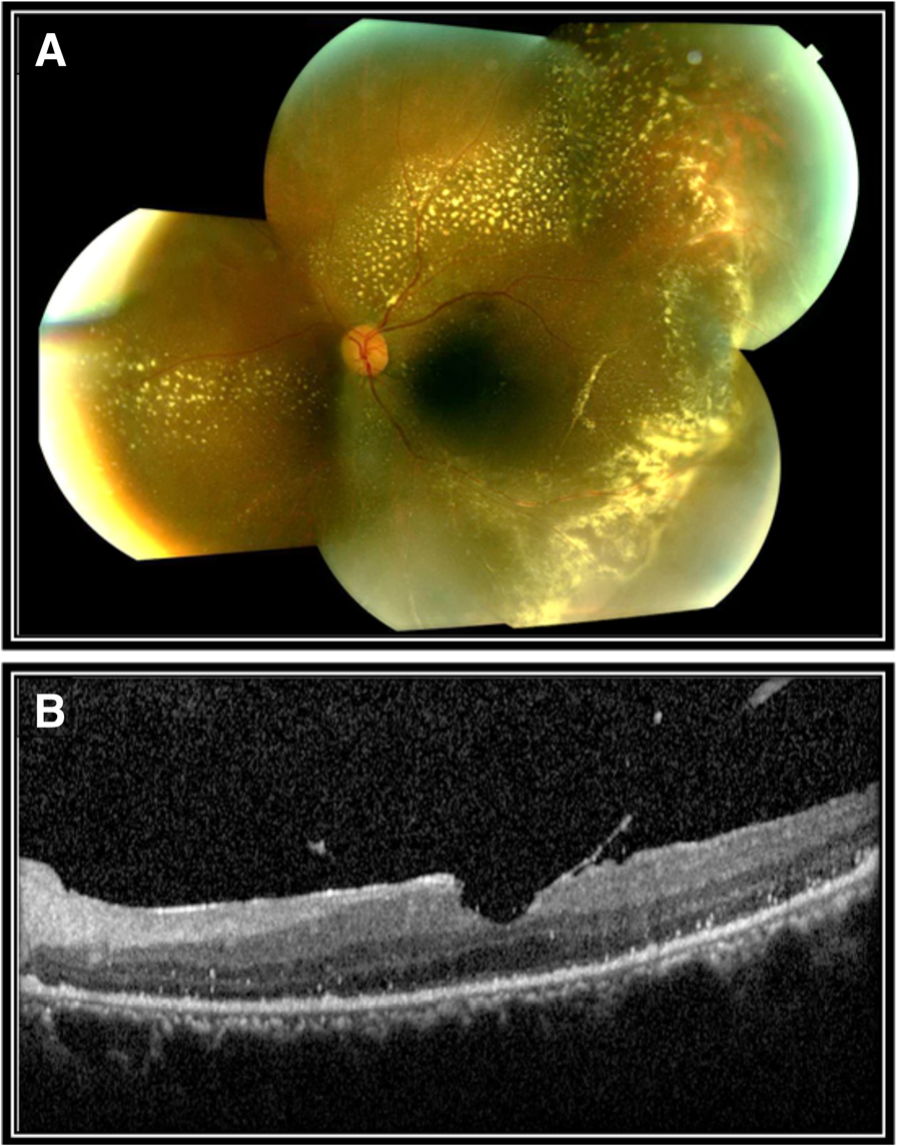
Post-operative fundus photography and optical coherence tomography of the left eye. Left eye status post pars plana vitrectomy and intravitreal injections with marked reduction of exudative retinal detachment and subretinal fluid with the presence of diffuse peripheral exudation as seen on fundus photography montage (**a**) and OCT (**b**)

The patient underwent intrathecal methotrexate and rituximab, cyclophosphamide, doxorubicin, vincristine, and prednisone (R-CHOP) with simultaneous intravitreal methotrexate (0.4 mg/0.1 ml) and rituximab (1 mg/0.1 ml). After five injections of methotrexate and four injections of rituximab, there was a complete resolution of the exudative retinal detachment as well as resolution of subretinal infiltrate and hemorrhage as seen on fundus photos and OCT (Fig. 6) and improvement in best corrected visual acuity to 20/200.

## Discussion

Our case represents a rare presentation of intraocular VRL associated with systemic lymphoma. In the current report, ocular symptoms preceded axillary lymph node diagnosis of DLBCL, demonstrating a rare case in the literature where intraocular manifestations were the first indications of systemic disease. It is unclear whether it is best to term this VRL as primary or secondary, because although the diagnosis of systemic lymphoma preceded the diagnosis of VRL, the ocular symptoms preceded systemic symptoms and diagnosis by 2 years. In general, intraocular lymphomas are rare. The vast majority of vitreoretinal lymphomas are primary (PVRL) and are categorized as a subset of primary CNS lymphoma (PCNSL) [3]. Secondary ocular lymphomas associated with systemic findings, however, are classically found to have choroidal infiltration (rather than vitreoretinal) due to hematogenous spread [2, 3].

The existence of SVRL is of clinical significance as it can masquerade as PVRL/PCNSL along with other vitreoretinal diseases. In cases of PVRL, the central nervous system is involved in 50–80% of patients, and therefore, the diagnostic focus is primarily intraocular and intracranial [1, 7]. In recent studies, SVRL associated with systemic lymphoma was found in 5–28% of patients after diagnostic vitrectomy [1, 3, 8]. Therefore, the lower prevalence of SRVL can often mislead clinicians into suspecting retinitis or vasculitis in the setting of immunosuppression from systemic lymphoma and chemotherapy, resulting in a delay of appropriate diagnosis and treatment [4, 6]. Among these reports, only a single other case was identified where ocular manifestations were the presenting signs of an undiagnosed systemic DLBCL. As such, in patients with recurrent vitreous hemorrhage without a clear etiology, as in our case, clinical suspicion for PVRL and SVRL may be warranted.

Intraocular lymphoma is diagnosed histologically with immunohistochemistry, flow cytometry, and PCR analysis [2]. Typically, intraocular lymphoma is of the non-germinal center B cell, or activated B cell (ABC), subtype [2]. Immunoprofiling usually demonstrates positive CD20, PAX5, CD79, BCL6, and BCL2, and an absence of CD10 and other plasma cell markers [2, 3, 5]. Our patient was classified by his oncologist as the ABC phenotype and analysis demonstrated positive CD19 and CD20, as well as negative CD10 on flow cytometry. IGH gene rearrangement analysis was positive, which is typical for vitreoretinal DLBCL [3, 8]. Karakawa et al. have suggested that the high correlation between SVRL and systemic ABC DLBCL may indicate a specific tropism for intraocular tissues [8].

The treatment for VRL is not well studied. In general, ABC DLBCL subtype has a poorer prognosis compared to germinal center B cell-like subtype (GCB) [9]. Current treatment for systemic ABC DLBCL is R-CHOP, although evidence has shown relapse in 40% of patients thereby spurring on newer frontline treatments targeting B cell pathways such as bortezomib or pomalidomide [9, 10]. For vitreoretinal DLBCL, there is no universally accepted treatment. Typical methods include a combination of local, regional, or systemic approaches. In prior published case reports, all patients received local therapy of either intravitreal methotrexate, rituximab, or both. Most patients also received local whole-brain and/or ocular radiation therapy, and only some underwent systemic chemotherapy plus intrathecal methotrexate. Our patient was treated with intravitreal injections of rituximab and methotrexate combined with systemic therapy with intrathecal methotrexate and R-CHOP.

Prognosis of PVRL has shown 3-year overall survival rates of 75–86% with treatment [8, 11]. The paucity of literature makes establishing prognosis for SVRL very difficult. Of the 9 patients included in 4 different case studies, 4 patients demonstrated overall survival time ranging from 14 to 62 months, while the remaining 55% were presently alive at the time of writing although the length of survival was unreportable due to lack of documentation [3–6].

## Conclusion

Although VRL is rare, ocular involvement preceding systemic involvement is even rarer. As seen in our case, recurrent vitreous hemorrhage without a clear underlying etiology may warrant some clinical suspicion for VRL. Its association with systemic lymphoma necessitates thorough and rapid intraocular, CNS, and systemic evaluation in patients. Treatment of VRL is not well established due to a lack of evidence-based studies for this uncommon condition. A combination of local, regional, and systemic therapies along with careful lifelong surveillance is recommended.

## Data Availability

Not applicable

## Abbreviations

ABC: Activated B cell subtype
CNS: Central nervous system
DLBCL: Diffuse large B cell lymphoma
FA: Fluorescein angiography
GCB: Germinal center B cell-like subtype
ICG: Indocyanine green
OCT: Optical coherence tomography
PCNSL: Primary CNS lymphoma
PVRL: Primary VRL
R-CHOP: Rituximab, cyclophosphamide, hydroxydaunorubicin, vincristine, prednisone
SVRL: Secondary VRL
VRL: Vitreoretinal lymphoma
